# The effects of Latino Dance intervention on academic and general self-efficacy with left-behind children: An experimental study in China

**DOI:** 10.3389/fpsyg.2023.1107233

**Published:** 2023-05-02

**Authors:** Zhenqian Zhou, Yutao Zhou, Francesco Vincenzo Ferraro, Andy Hooton, Chris Ribchester

**Affiliations:** ^1^School of Artistic Sport, Hunan Agricultural University, Changsha, China; ^2^Physical Education College, Hunan University of Technology, Zhuzhou, China; ^3^School of Human Sciences, University of Derby, Derby, United Kingdom; ^4^Pedagogical Innovation Enhancement Research Team, University of Derby, Derby, United Kingdom

**Keywords:** Latin America Dance, left-behind children, general self-efficacy, Student’s academic self-efficacy, self-esteem

## Abstract

**Introduction:**

Although there is considerable research indicating that physical exercise and dance can strengthen children’s self-efficacy, and children’s self-efficacy can predict students’ academic achievement at a wide range of academic levels. Few studies have been conducted using Latino Dance to improve self-efficacy in Left-Behind Children (LBC), especially the two aspects of student academic self-efficacy and general self-efficacy, while the mediator role of self-esteem between student academic self-efficacy and general self-efficacy has been less explored in previous research.

**Methods:**

This study proposed to examine Latino Dance interventions to improve general self-efficacy and students’ academic self-efficacy among LBC students in rural areas to boost students’ academic performance, and the research team hypothesised that general self-efficacy, students’ academic self-efficacy and self-esteem would improve following the intervention and that outcomes will have a significant positive correlation as students’ self-esteem can mediate both their academic self-efficacy and their general self-efficacy. Dates were collected from 305 LBCs children (160 boys and 145 girls) from 6 left-behind schools in Hunan province, China. Ralf Schwarzer’s general self-efficacy scale, Morgan-Jinks Student academic Self-Efficacy Scale, and Rosenberg’s self-esteem scale were administered to LBCs between September 2020 and January 2022.

**Results:**

The results revealed that the Latino Dance intervention significantly increased the LBC student’ academic self-efficacy and general self-efficacy, which also involved a positive effect on the three sub-dimensions (talent, context, and effort) of students’ academic self-efficacy. Further, multiple linear regression analysis confirmed that self-esteem (positive esteem/self-deprecation) acted as a partial mediator between student academic self-efficacy and general self-efficacy; perceived self-esteem played a mediating role between them.

**Discussion:**

This study filled a gap in the literature concerning the psychological reinforcement effect of Latino Dance on LBC groups and demonstrated that Latino Dance improved the student’ academic self-efficacy and general self-efficacy among the LBCs. Our results suggest that Latino Dance can be beneficial for LBC in school by including Latino Dance in Physical Education or Art courses and improving students’ self-esteem may lead to an increase in student academic self-efficacy as well as general self-efficacy, thereby improving and enhancing the learning of LBCs.

## 1. Introduction

Since nearly two decades of economic reform in China (Wang and Sun, 2021), many parents from rural areas moved to industrialised cities looking for job opportunities (Gu, 2022). Research shows that this gentrification has a significant impact on younger generations producing the phenomena of left-behind children (LBC), defined as children who cannot move to the cities for longer than six months (Li et al., 2020; Zhang et al., 2021) and are left in the care of grandparents or rural communities (Zhang et al., 2020; Chen et al., 2022).

In particular, previous studies showed that LBCs have a higher level of school dropouts (Li and Hu, 2021; Wang Haining, 2021) due to the lack of parental supervision and family education (Li and Hu, 2021), which heavily impacts LBCs in various aspects of their lives, such as their academic achievement (Song et al., 2018; Wang et al., 2019; Gu, 2022), future life opportunities (Ge et al., 2019), as well as they were suffered from mental health conditions (Chen et al., 2022; Zhou et al., 2022), such as high suicidal thoughts (Zhou et al., 2022), high risk of delayed neurodevelopment (Li et al., 2021), depressive symptoms (Ma et al., 2022), antisocial behavioural (Liu et al., 2022), lower self-esteem (Zhou et al., 2022) and lower self-efficacy (Liang et al., 2018). He et al. (2020) and Gao et al. (2022) highlighted that improving the self-efficacy and academic performance of LBCs has become a widely discussed topic in academia recently.

Based on previous pedagogic research, we can identify general and academic self-efficacy as two significant psychological indicators that predict students’ academic performance and success (Ohrtman and Preston, 2014; Morales-Rodríguez and Pérez-Mármol, 2019). General self-efficacy refers to people’s belief that they will be able to perform well under certain conditions (Evers et al., 2002). Usher and Pajares (2008) highlighted in a literature review that general self-efficacy has received much attention in educational research as it impacts students’ academic performance at a wide range of academic levels. Also, Pallathadka (2020) found that general self-efficacy has a significant correlation with self-esteem and self-esteem as a psychological condition that represents a sense of integrity in understanding personal capabilities and self-worth (Pyszczynski et al., 2004), which can positively predicted academic performance (Román et al., 2008; Saha and Tamanna, 2018). Additionally, students’ academic self-efficacy refers to a person’s belief that he or she will be able to meet their academic environment’s demands (Fife et al., 2011). Arslan (2012) noted that students who lower academic self-efficacy are less eager to learn, have difficulty concentrating at school and have difficulty overcoming challenges at school, and Fan and Williams (2010) noted that students with higher academic self-efficacy were more engaged and enthusiastic about their learning. For the aim of the following document, self-worth is defined as a sense of personal value as Merriam-Webster (2022), whilst self-esteem is defined as a sense of self-worth, which is crucial for understanding people’s well-being and success (Monteiro et al., 2022). Research shows that self-esteem positively predicted academic achievement in various study contexts, such as in university students (Román et al., 2008) and school adolescents (Saha and Tamanna, 2018). Also, it is known to play a role in mediating the relationship between parent–child relationships and academic stress (Mulyadi et al., 2016), as well as strong and positive relationships between teacher support and academic performance (Liu et al., 2019).

The recent research of Barczak and Bednarek (2020) found that Latino Dance (a kind of partner dance originating in Latin America that includes dances such as the cha-cha-cha, rumba, samba, jive, and paso doble) can improve general self-efficacy among young adults. Several studies have shown that Latino Dance plays an important role in the treatment of human body health and mental well-being. In particular, Latino Dance diminishes anxiety (Hong-Gang Shan, 2011), self-accusation tendency, loneliness (Hong-Gang Shan, 2011), and improves self-efficacy (Duan Hui and Junyu, 2014), students’ self-esteem (Jie, 2007).

The following study aims to examine if Latino Dance interventions improve general self-efficacy and students’ academic self-efficacy among LBC students in rural areas and if Latino Dance will boost students’ academic performance.

The research team hypothesised that general self-efficacy, students’ academic self-efficacy and self-esteem would improve following the intervention and that outcomes would have a significant positive correlation as students’ self-esteem can mediate both their academic self-efficacy and their general self-efficacy.

## 2. Methods

### 2.1. Participants

During September 2020 and January 2022, a cross-sectional study was conducted in six randomly selected primary schools in Hunan province (southern China) from a list given by the government-sponsored Hunan Women and Children’s Federation. After receiving permission from each school principal, 367 LBC students (from year four to year six) under the researcher’s guidance voluntarily registered (parent/guardian consent is required) to participate in a Latino Dance training intervention.

Before and after the intervention, all participants were administered self-reported questionnaires by trained research assistants. Information, including demographic characteristics, opinions about their experiences within the study, and mental health were collected, whilst three questionaries including the Individual General Self-Efficacy Scale, Morgan-Jinks Student Efficacy Scale (Children’s Perceived Academic Self-Efficacy Scale), and Self-Esteem Scale were used to meet the study aim.

### 2.2. Questionnaires

#### 2.2.1. Demographic questionnaire and general self-efficacy scale

Using the demographic questionnaire, LBCs’ age and sex were identified and are shown in Table 1. Another type is the survey questionnaire, which is the “general self-efficacy scale” (Schwarzer and Jerusalem, 1995), Schwarzer and Jerusalem developed in 1981, which is a 10-item psychometric scale that rates optimistic self-beliefs about the ability to cope with difficult situations in life. Each item is awarded a four-point (1–4) value ranging from “Not at all true” to “Exactly true,” including “Not at all true,” “Hardly true,” “Moderately true,” and “Definitely true.” During this study, we asked the participants to provide us with their responses as accurately as possible, reminding them that all the information they provided would be treated confidentially.

**Table 1:** Demographic characteristics of the respondents (*N* = 305).

Characteristics	Items	Frequency	Percentage (%)
Sex	Male	160	52.5
	Female	145	47.5
Age	9	109	35.7
	10	50	16.4
	11	52	17.0
	12	72	23.6
	13	22	7.3

#### 2.2.2. Morgan-Jinks academic self-efficacy scale

Jinks and Morgan (1999) Children’s Perceived Academic Self-Efficacy Scale was used to measuring academic self-efficacy in left-behind children, which is intended to provide practitioners with insight into children’s self-efficacy beliefs about their academic success while in school. Morgan and Jinks developed it in 1999. Response options are presented on a four-point Likert scale, using informal language that reflects the manner in which children speak. On the scale, there are 30 items ranging from fully agree to completely disagree, and the scale was divided into three sub-dimensions based on a student’s talents, efforts, and context.

#### 2.2.3. Rosenberg’s self-esteem scale

The Rosenberg’s self-esteem scale (Rosenberg, 1979) assesses a person’s self-worth and is designed to evaluate a child’s or adolescent’s opinions about their own worth. Teenagers using Rosenberg’s self-esteem scale are able to assess both positive and negative feelings about themselves, thus estimating their sense of self-worth (Rosenberg, 1965; Cheng and Hamid, 1995). The scale consists of 10 items: positive esteem (5 items) and self-deprecation (5 items). Examples of items include the Positive esteem, e.g., “in general, I am satisfied with myself,” and the self-deprecation, e.g., “at times I think I am no good at all.” Reverse coding was applied to items with a negative value. Each item is answered using a 4-point Likert scale (1–4), ranging from 1 (strongly disagree) to 4 (strongly agree), including strongly disagree, disagree, agree, and strongly agree. A scale of 10–40 was used, where higher scores indicate greater self-esteem.

### 2.3. Intervention

Between September 2020 and January 2022, Latino Dance interventions were scheduled in 96 lessons over three semesters, 32 lessons each semester, twice per week, and 1.5 h per lesson, 144 h in total. (Semester one is from September 2020 to January 2021, Semester two is from February to June 2021, and Semester three is from September 2021 to January 2022).

Students in six schools were taught the same two sub-types of Latino Dance: Cha Cha Cha and Samba. There are four major components of this course: basic dance movement, solo and couple dance routines, music understanding, and dance movements-images. During the three semesters, the LBCs learned all dance routines and were able to perform on stage at the end, also at the end of each academic year, each school did a staged choreographed performance as a way to evaluate the quality of the teacher’s instruction. Finally, the top 3 teams representing their school were offered an opportunity to perform on national TV, and everybody appreciated this activity which can show a proud moment for the children’s families.

### 2.4. Procedure

**Figure 1:**
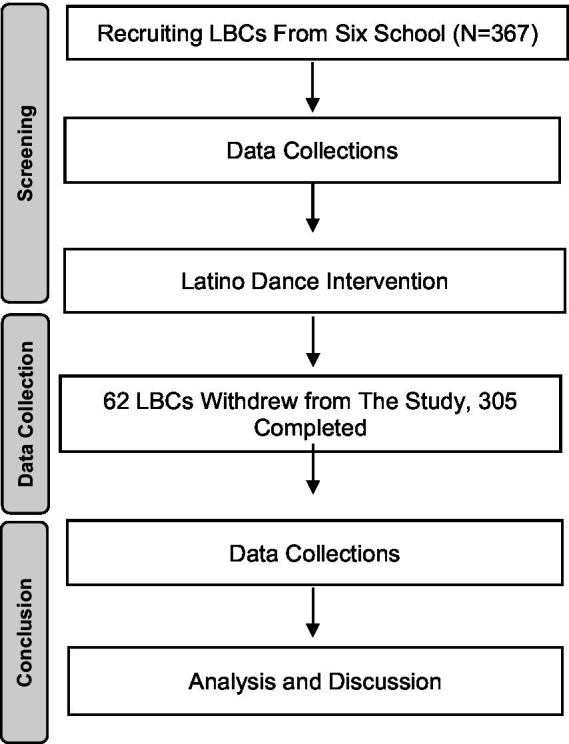
PRISMA flow diagram displaying research procedure.

A group of 18 Latino Dance teachers qualified to teach were selected before the research intervention (the teachers had a minimum of 3 years of experience in teaching Latino Dance and were an equal number of 9 male and 9 female). A random drawing was conducted to divide them into six groups and assign them to six LBC primary schools with three dance teachers each. In parallel, three additional researchers (PhD level) with backgrounds in psychology were trained by this researcher’s team in advance to help collect data as well as to assist the students in completing the questionnaire. Three standardised questionnaires were administered to all LBCs in six schools pertaining to general self-efficacy, self-esteem, and student academic self-efficacy at the start and end of the intervention study (See Figure 1).

### 2.5. Statistical methods

To meet the study aim (i.e., examining Latino Dance interventions to improve general self-efficacy and students’ academic self-efficacy among LBC students in rural areas to boost students’ academic performance). Data reliability and validity of the questionnaires were tested using Cronbach’s alpha (*α*) and Kaiser-Meyer-Olkin (KMO) coefficients, and the quantitative data were assessed by Paired *T*-tests to compare various demographic variables and pre- vs. post-interventional treatment. The results of the measured values were stated as mean ± standard deviation. Meanwhile, Pearson correlation analysis was used to determine correlations between the variables (LBC’s general self-Efficacy, self-Esteem, and student academic self-efficacy). Multiple linear regression was used to assess the role of the variables as mediators. The significance level was regarded as *p* < 0.05 for two-sided tests. SPSS 27.0 was used for the statistical analysis. Finally, mediation analyses were conducted to investigate whether self-esteem acted as a mediator between academic self-efficacy and general self-efficacy among students. Pre-intervention data was used, as it had the potential to better reflect the true mental state of LBCs, after the data collection had ended.

## 3. Results

In this study, 17% (62 of 367) LBCs withdrew from the intervention because several reasons, including they thought the dance was boring, feeling uncomfortable when dancing and being shy. A total of 305 participants aged 9 to 13 years completed all lessons. Among them, one hundred sixty (160) boys (52.50%) and one hundred forty-five (145) girls (47.50%) in the average age group of *M* = 10.502, SD = 1.367.

### 3.1. Reliability and validity of scales

The Cronbach’s alpha (*α*) coefficient and Kaiser-Meyer-Olkin (KMO) coefficient were used to test the internal consistency of the overall scales, and this study showed good reliability and validity. A general threshold for Cronbach’s alpha (*α*) coefficient is generally accepted as 0.9 ≤ *α* is deemed excellent, 0.7 ≤ *α* < 0.9 good, 0.6 ≤ *α* < 0.7 acceptable (Kulthanan et al., 2019); while with Kaiser-Meyer-Olkin (KMO) coefficient states that 0.9 ≤ KMO is indicated very suitable, 0.8 ≤ KMO < 0.9 suitable, 0.7 ≤ KMO < 0.8 average suitability, 0.6 ≤ KMO < 0.7 mediocrely suitable, 0.5 ≤ KMO < 0.6 barely suitable, and KMO < 0.5 not suitable (Li, 2021). In this research, Table 2 shows that in Schwarzer and Jerusalem’s general self-efficacy scale, Cronbach’s alpha coefficient in this study is 0.91, and the KMO coefficient is 0.92; For Morgan and Jink’s children’s perceived academic self-efficacy scale, Cronbach’s alpha coefficient and KMO coefficient of this study are both 0.79 and 0.82, respectively. For Rosenberg’s self-esteem scale, Cronbach’s alpha coefficient is 0.77, while the KMO coefficient is 0.85.

**Table 2:** Reliability and validity of scales.

Scale	Cronbach’s α	Kaiser-Meyer-Olkin (KMO)
General self-efficacy scale	0.905	0.916
Students’ academic self-efficacy scale	0.788	0.825
Self-esteem scale	0.768	0.854

### 3.2. Impact of Latino Dance among left-behind children

According to the post-test statistics, General Self-Efficacy, Self-Esteem, and Student academic self-efficacy scores were significantly above the pre-test levels (*p* < 0.01), which shows in Table 3 and there was a statistically significant increase in the same gender group between pre-test and post-test (*p* < 0.01). It was demonstrated that Latino Dance has a significant impact on all LBC’s psychological variables of General Self-Efficacy, Self-Esteem, and Student academic self-efficacy.

**Table 3:** Pre-test and post-test of Latino Dance Intervention (*N* = 305).

Group	Paired statistics (Mean ± SD)		Paired mean	*T*	*p*
	Pre-test	Post-test			
Academic self-efficacy	62.91 ± 7.48	90.35 ± 10.08	−27.44	−38.223	0.000^**^
Male	63.24 ± 7.43	91.12 ± 9.82	−27.881	−27.845	0.000^**^
Female	62.56 ± 7.56	89.50 ± 10.34	−26.945	−26.133	0.000^**^
Self-esteem	15.59 ± 2.93	29.19 ± 4.48	−13.60	−44.782	0.000^**^
Male	15.46 ± 2.77	29.31 ± 4.75	−13.844	−31.847	0.000^**^
Female	15.73 ± 3.09	29.06 ± 4.18	−13.324	−31.568	0.000^**^
General self-efficacy	17.90 ± 4.83	25.55 ± 6.08	−7.65	−16.972	0.000^**^
Male	17.57 ± 4.72	25.43 ± 5.86	−7.862	−13.214	0.000^**^
Female	18.27 ± 4.94	25.68 ± 6.34	−7.407	−10.814	0.000^**^

Table shows the general means (Mean), standard deviations (SD). Paired *T*-tests to exam differences Pre-, Post-test.

^*^ Indicates a weak significant relationship between the variables.

^**^ Indicates a strong significant relationship between the variables.

Based on this finding, Table 4 demonstrates that Latino Dance also has a positive effect on the sub-dimensions of Student academic self-efficacy, including variables of talent, context, and effort. The statistical data are all significantly higher than before the intervention (*p* < 0.01). Meanwhile, Table 5 shows that before Latino Dance intervention on LBCs’ student academic self-efficacy, there were no statistically significant differences between 9 and 13-year-old students (*p* > 0.05). Also, there was no statistically significant difference in academic self-efficacy between 9 and 13-year-old students after the Latino Dance intervention (*p* > 0.05), despite this, the overall score rose on the post-test. In addition, although students’ self-esteem and general self-efficacy differed significantly between age groups (9–13) before the Latino Dance intervention, and after the Latino Dance intervention, data on self-esteem and general self-efficacy did not differ significantly between age groups (*p* > 0.05).

**Table 4:** Pre-test and Post-test of Sub-Dimension in student academic self-efficacy (*N* = 305).

Group	Paired statistics (Mean ± SD)		Paired mean	*T*	*p*
	Pre-test	Post-test			
Talent Factor	27.67 ± 4.27	39.28 ± 4.70	−11.60	−31.358	0.000^**^
Male	27.86 ± 4.27	39.62 ± 4.63	−11.762	−22.723	0.000^**^
Female	27.47 ± 4.28	38.90 ± 4.76	−11.428	−21.554	0.000^**^
Context Factor	26.58 ± 3.48	39.42 ± 5.75	−12.84	−33.415	0.000^**^
Male	26.66 ± 3.56	39.95 ± 5.59	−13.288	−25.126	0.000^**^
Female	26.48 ± 3.41	38.83 ± 5.89	−12.345	−22.114	0.000^**^
Effort Factor	8.67 ± 1.84	11.66 ± 1.67	−2.99	−21.366	0.000^**^
Male	8.72 ± 1.93	11.55 ± 1.76	−2.831	−13.815	0.000^**^
Female	8.61 ± 1.75	11.78 ± 1.55	−3.172	−16.821	0.000^**^

Table shows the general means (Mean), standard deviations (SD). Paired *T*-tests to exam differences Pre- and Post-test.

^*^ Indicates a weak significant relationship between the variables.

^**^ Indicates a strong significant relationship between the variables.

**Table 5:** Pre-test and Post-test of different age group (*N* = 305).

		Age (Mean ± SD)					*F*	*p*
		9.0 (*n* = 109)	10.0 (*n* = 50)	11.0 (*n* = 52)	12.0 (*n* = 72)	13.0 (*n* = 22)		
Academic self-efficacy	Pre-test	62.65 ± 7.10	63.54 ± 10.11	61.92 ± 4.48	63.58 ± 7.94	62.95 ± 6.81	0.490	0.743
	Post-test	89.63 ± 8.83	88.90 ± 11.21	90.62 ± 11.01	91.97 ± 10.50	91.27 ± 9.64	0.916	0.455
Self-esteem	Pre-test	15.48 ± 2.99	16.44 ± 2.56	15.96 ± 3.16	15.32 ± 2.92	14.23 ± 2.31	2.713	0.030*
	Post-test	28.74 ± 4.53	29.16 ± 4.41	29.52 ± 4.07	29.78 ± 4.42	28.73 ± 5.58	0.707	0.588
General self-efficacy	Pre-test	18.32 ± 4.97	17.78 ± 4.31	16.10 ± 4.50	18.69 ± 4.85	17.77 ± 5.22	2.571	0.038*
	Post-test	25.78 ± 5.91	25.24 ± 6.41	25.17 ± 6.31	26.00 ± 6.27	24.50 ± 5.30	0.380	0.823

Table shows the general means (Mean), standard deviations (SD). One-way analysis of variance (ANOVA) to examine the differences in age between Pre- and Post-Tests.

^*^ Indicates a weak significant relationship between the variables.

^**^ Indicates a strong significant relationship between the variables.

### 3.3. Correlation between academic/general self-efficacy and self-esteem

Table 6 shows the correlational and statistical data for the main variables, including student academic self-efficacy, general self-efficacy, and self-esteem. As shown in Table 6, the correlation analysis evaluated the correlation between the five items: Student academic self-efficacy, with its three sub-dimensions (Talent, Context, and Effort), Self-Esteem, and General Self-Efficacy. *R* values have been used to identify strong or weak correlations between each variable and indicate the degree of correlation. According to the specific analysis: student academic self-efficacy is strongly correlated with self-esteem and general self-efficacy, with a significant statistical correlation (*p* < 0.01). Additionally, Student academic self-efficacy was significantly and positively associated with the three sub-dimensions of Talent, Context, and Effort (*p* < 0.01). Within the three sub-dimensions of Student academic self-efficacy, the variables Talent and Context show significant positive correlations with Self-Esteem and General Self-Efficacy (*p* < 0.01). However, the variable “effort” does not show a significant correlation between Self-Esteem and General Self-Efficacy (*p* > 0.05), despite the global score of Student academic self-efficacy representing a significant positive correlation with these two variables. Student self-esteem shows a high correlation with Student academic self-efficacy and General Self-Efficacy (*p* < 0.01). The relationship between general self-efficacy and self-esteem and student academic self-efficacy was significantly and positively significant (*p* < 0.01).

**Table 6:** Descriptive statistics and pearson correlation coefficient table (*n* = 305).

	Mean	SD	1	2	3	4	5	6
Age	10.502	1.367						
1. Academic self-efficacy	90.351	10.084	1					
2. Talent factor	39.275	4.696	0.876**	1				
3. Context factor	39.416	5.750	0.905**	0.629**	1			
4. Effort factor	11.659	1.667	0.459**	0.316**	0.256**	1		
5. Self-esteem	29.187	4.483	0.195**	0.180**	0.207**	−0.039	1	
6. General self-efficacy	25.548	6.084	0.232**	0.179**	0.258**	0.007	0.240**	1

All tests were two-tailed. This table shows the general means (Mean), standard deviations (SD), and correlations of the six major variables.

^*^ Indicates a weak significant relationship between the variables.

^**^ Indicates a strong significant relationship between the variables.

### 3.4. Mediator role in self-esteem

**Figure 2:**
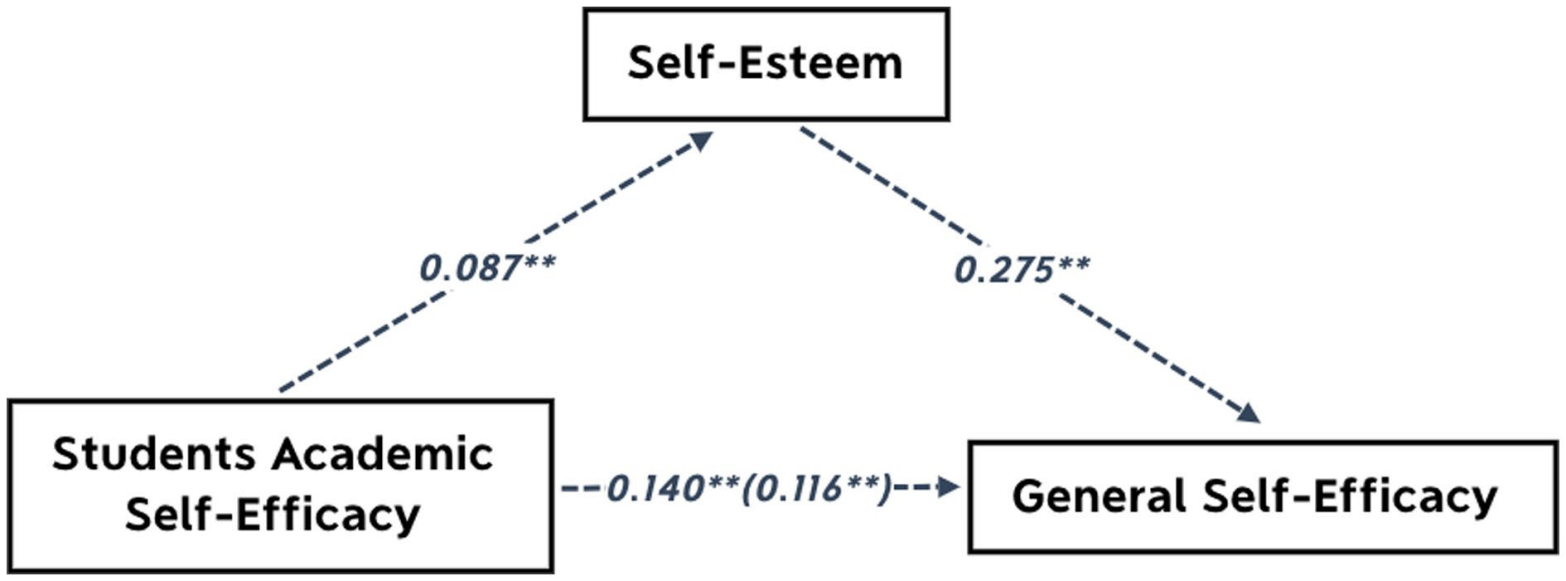
Model of meditation effect (indirect effects), **Significant (*p* < 0.01).

A linear regression analysis was conducted to determine the mediating effect (See Figure 2).

This study hypothesised that self-esteem served as a mediator, student academic self-efficacy served as an independent variable, and general self-efficacy served as a dependent variable; then considered general self-efficacy as the dependent variable and student academic self-efficacy as the prediction variable. As shows in Table 7, study results indicated that student academic self-efficacy is an effective predictor of general self-efficacy (*β* = 0.232, *p* < 0.01). Consequently, study employed self-esteem as the dependent variable and student academic self-efficacy as the predictor variable. It was found that student academic self-efficacy had an effect on the prediction of self-esteem (*β* = 0.195, *p* < 0.01).

**Table 7:** Mediator effect of self-esteem on student academic self-efficacy and general self-efficacy (*N* = 305).

Step	Independent variable	Dependent variable	*β*	*T*	*R* ^2^	Δ*R*^2^	*F*
1	Academic self-efficacy	General self-efficacy	0.232	4.148	0.054	0.051	17.207^**^
2	Academic self-efficacy	Self-esteem	0.195	3.465	0.038	0.035	12.008^**^
3	Academic self-efficacy	General self-efficacy	0.192	3.440	0.093	0.087	15.541^**^
	Self-esteem		0.203	3.631			

Model 1, Regression model of “Academic self-efficacy” to “General Self-Efficacy”.

Model 2, Regression model of “Academic self-efficacy” to “Self-Esteem”.

Model 3, Regression model of “Academic self-efficacy” with the mediating variable “Self-Esteem” to “General Self-Efficacy”.

^*^ Indicates a weak significant relationship between the variables.

^**^ Indicates a strong significant relationship between the variables.

In addition, as reported in Tables 7, 8 that use the score of general self-efficacy as the dependent variable and students’ academic self-efficacy and self-esteem as the independent variables, the results of the regression analysis revealed that when the self-esteem variable was included, the prediction of student academic self-efficacy for general self-efficacy decreased (Table 8 displays B from 0.140 dropped to 0.116), but was still significant (*β* = 0.192, *p* < 0.01). As well the results show that self-esteem can effectively predict general self-efficacy (*β* = 0.203, *p* < 0.01). Moreover, the result shows that the indirect effect of self-esteem between Student academic self-efficacy and General Self-Efficacy was significant for 95% Boot CI (the interval is 0.010 ~ 0.081, does not include 0), and the mediation effect was 0.024. A 17.085% indirect effect was accounted for in the total effect. The above finding indicated that self-esteem acted as a partial mediator between Student academic self-efficacy and General Self-Efficacy. Perceived self-esteem plays a mediator role.

**Table 8:** Mediation effect results.

Item	Result	B(c) TE	a	b	a × b ME	B(c′) DE	a × b (95% Boot CI)	Formula	Effect ratio
Academic self-efficacy	Partial mediator	0.140**	0.087**	0.275**	0.024	0.116**	0.010~0.081	a × b/c	17.085%
Self-esteem									
General Self-Efficacy									

“B(c)”: Represents the regression coefficient of “Academic self-efficacy” to “General Self-Efficacy” (no mediator Self-Esteem in the model), i.e., Total Effect (TE).

“B(c′)”: Represents the regression coefficient of “Academic self-efficacy” to “General Self-Efficacy” (included mediator Self-Esteem in the model), i.e., Direct Effect (DE).

“a”: Represents the regression coefficient of “Academic self-efficacy” to “Self-Esteem”.

“b”: Represents the regression coefficient “Self-Esteem” to “General Self-Efficacy”.

“a × b”: Represents the product of a and b, i.e., Mediation effect (ME). 95% Boot CI represents the 95% confidence interval calculated by Bootstrap sampling, if the interval does not include 0, it means significant.

^*^ Indicates a weak significant relationship between the variables.

^**^ Indicates a strong significant relationship between the variables.

## 4. Discussion

### 4.1. Impact of Latino dance among left-behind children

The result accepted the research hypothesis that Latino Dance intervention could effectively improve general self-efficacy, students’ academic self-efficacy and self-esteem among LBC students in Chinese rural areas to boost students’ academic performance.

In this research, significant improvements were seen in each psychometric test variable, including the LBC’s general self-efficacy, self-esteem, and student academic self-efficacy. The result shows that the Latino Dance intervention significantly improved three sub-dimensions of students’ academic self-efficacy: talent, learning context, and effort. This suggests that Latino Dance can positively affect student academic self-efficacy, general self-efficacy, and self-esteem of LBC in rural areas, which matches the research aim of this study.

Our results also show that LBCs’ self-esteem and general self-efficacy levels regarding before Latino Dance intervention (following the pre-test) showed significant differences between the age groups (9–13) (*p* < 0.05). However, after the intervention, there was no significant difference in self-esteem and general self-efficacy between age groups (*p* > 0.05). This result may be caused by the fact that before the intervention, there were significant disparities in the children’s life experiences, family circumstances, and living conditions, leading to significant differences in their self-esteem and general self-efficacy between the ages of 9 and 13. After the intervention, general self-efficacy, students’ academic self-efficacy, and self-esteem increased significantly, and they all achieved an average level.

In previous research, Smith (2014) examined the effects of salsa lessons on the general self-efficacy level in 57 non-regular exercisers between 18 and 45 years of age who exercised less than 30 min per session, three times per week or less. The results indicated that salsa dance positively impacted adults’ general self-efficacy. Also, Arman and Turkmen (2021) found that a dance therapy course could effectively increase academic motivation and dance-related self-efficacy in 102 university students (21.33 ± 2.92 years). Further, Schwender et al. (2018) systematically reviewed the effects of dance interventions on children, adolescents, and adults, which shows that dance is an effective method and has positive effects on participant self-esteem and self-efficacy. As stated above, although several prior studies found that dance-related activities had a significant and positive impact on general self-efficacy, academic motivation, and self-esteem, the current research shows that Latino Dance increased LBC’s general self-efficacy, self-esteem, and student academic self-efficacy significantly statistically. This study suggests that Latino Dance can be beneficial for LBCs to adopt in school as part of Physical Education or Art courses.

### 4.2. Correlation between academic/general self-efficacy and self-esteem

The results also accepted the research hypothesis that general self-efficacy, students’ academic self-efficacy and self-esteem have a significant positive correlation among LBC students. According to the findings of this study, student academic self-efficacy presents a significant positive correlation with self-esteem and general self-efficacy, this result is consistent with Luo et al. (2022) and Zhang et al. (2022) funding.

Luo et al. (2022) investigated 2,473 Chinese college students (1,166 females and 1,307 males) and found that there were significant relationships between self-esteem and student academic self-efficacy, and high academic self-efficacy was also associated with academic performance, which may predict high self-esteem.

Whilst Zhang et al. (2022) investigated 1,502 Chinese full-time undergraduate students from 10 universities in Beijing and found that self-esteem and general self-efficacy were significantly and positively correlated.

On the other hand, the results indicate that the two sub-dimensions (talent and context) of LBC’s academic self-efficacy have a significant correlation with LBC’s self-esteem and LBC’s general self-efficacy. Among them (talent and context), the context dimension is highly correlated with students’ academic self-efficacy and also is highly correlated with “self-esteem” and “general self-efficacy,” which was consistent with the research of Nurmina (2017) and Usán Supervía and Alberto (2021).

Nurmina (2017) found that the context dimension of family involvement has a significant impact on children’s academic self-efficacy after researching 97 children from 5 to 6 grades of elementary school.

Whilst Usán Supervía and Alberto (2021) stated that different emotional and motivational states significantly affect students’ academic performance, also Usán Supervía and Alberto (2021) investigated 2,204 students (12–18 years) and found that general self-efficacy mediates the relationship between emotion regulation and academic performance. In contrast, the “effort dimension,” which is one of the subdimensions of students’ academic self-efficacy, did not show to be associated with self-esteem and general self-efficacy. However, Ferkany (2008) researched an attachment theoretic account of self-esteem, which shows that self-esteem is a crucial factor in children’s confidence, motivation, and ability to achieve educational goals. Therefore, there is a possibility that the current living environment of left-behind children causes them to lose confidence, which negatively affects their self-efforts to achieve high self-esteem levels.

Furthermore, the results also indicate that self-esteem and general self-efficacy can influence student academic performance, which is compatible with Pallathadka (2020) and Asakereh and Yousofi (2018). Pallathadka (2020) studied a group of 205 university students and found that general self-efficacy and self-esteem have significant connections and influence the student’s academic performance and achievement. Also, Asakereh and Yousofi (2018) investigated the relationship between general self-efficacy, self-esteem, and academic achievement in 132 “English as a foreign language (EFL)” students, showing significant correlations between EFL general self-efficacy, self-esteem, and positive influence students’ academic achievement.

### 4.3. Mediator role in self-esteem

Based on our findings, self-esteem played a partial intermediary role between student academic self-efficacy and general self-efficacy, which indicates that self-esteem has an intermediary effect on enhancing LBC’s general self-efficacy when it is enhanced by LBC’s academic self-efficacy. Therefore, the research hypothesis in this research has not been rejected. There are probably two reasons: Firstly, Frank et al. (2010) investigated 158 adolescents in school and reported that the two sub-dimensions of positive esteem and self-deprecation in self-esteem could impact and correlate with general self-efficacy. Also, Hashemi et al. (2017) investigated 212 patients with type 2 diabetes and found that self-esteem plays a positive mediating role in improving general self-efficacy. Secondly, through the development of predictive structural models, Soufi et al. (2014) found that self-esteem can predict students’ academic achievement. Also, Ugwuanyi et al. (2020) found that self-esteem had a significant impact on the mathematical achievement of students, based on data collected from 400 students in 16 secondary schools, which indirectly impacts academic self-efficacy. Further, Liu et al. (2019) based on investigating the relationship between self-esteem and migrant children’s academic achievement revealed that self-esteem (positive esteem and self-deprecation) mediated the positive relationship between teacher support and academic achievement. As stated above, although prior studies found that self-esteem had a mediation effect on students’ general self-efficacy and academic achievement, the current research also shows that self-esteem mediated LBC’s student academic self-efficacy and general self-efficacy; therefore, this study suggests that improving students’ self-esteem may lead to an increase in student academic self-efficacy as well as general self-efficacy thereby improving the learning of left-behind children.

## 5. Conclusion

The study purpose was to examine whether or not Latino Dance interventions improve general self-efficacy and students’ academic self-efficacy among LBC students in rural areas of China and if it improves students’ academic performance.

The results show that Latino Dance significantly improved the general self-efficacy, students’ academic self-efficacy, and self-esteem of all psychometric test factors in the LBC. Second, student academic self-efficacy shows a significant correlation with self-esteem and general self-efficacy. In addition, two subdimensions (talent and context) of student academic self-efficacy also show a significant correlation with self-esteem and general self-efficacy; however, the “effort” dimension of student academic self-efficacy did not appear to be related to self-esteem and general self-efficacy. The study also explored the mediation effect of self-esteem (positive esteem/self-deprecation) between student academic self-efficacy and general self-efficacy, and the findings showed that self-esteem played a partial intermediary role between student academic self-efficacy and general self-efficacy.

This study fills a gap in the literature regarding the psychological reinforcement effect of Latino Dance on LBC groups and examines the correlation relationship between general self-efficacy, self-esteem, and student academic performance as indicators of Latino Dance’s psychological reinforcement effect on LBC groups. This research suggests that Latino Dance can be beneficial for LBC in school by including Latino Dance in Physical Education or Art courses and improving students’ self-esteem may lead to an increase in student academic self-efficacy as well as general self-efficacy thereby improving and enhancing the learning of LBCs.

## 6. Limitations and future research directions

Several limitations accompany this study. This study examined the effects of Latino Dance (Cha Cha Cha and Samba) on the general/students’ academic self-efficacy of LBC, which considered the two styles as a whole intervention. It would be more valuable to explain the impacts of different dance styles, such as in this case, comparing Cha Cha Cha and Samba separately. Also, research suggests that Latino Dance can be beneficial for LBCs in improving students’ self-esteem, which may lead to an increase in student academic self-efficacy and general self-efficacy. However, the relationship between students’ self-esteem and academic performance has not yet been examined. Further studies could explore this link and shed light on how Latino Dance can potentially enhance academic outcomes (performance) for LBC students.

In addition, only three semesters (one and a half years) of intervention in Latino Dance (Cha Cha Cha and Samba) to measure general/students’ academic self-efficacy and self-esteem was carried out, thus a more comprehensive analysis of the long-term effects of our study is necessary. Further research should examine different dance styles for sustainable Latino Dance interventions and provide a wider range of psychological indicators, such as social anxiety and loneliness, to obtain more comprehensive results for research in Latino Dance intervention strategies.

## Data availability statement

The raw data supporting the conclusions of this article will be made available by the authors, without undue reservation.

## Ethics statement

The studies involving human participants were reviewed and approved by The Research Ethics Committee of Hunan Agricultural University. Written informed consent to participate in this study was provided by the participants' legal guardian/next of kin.

## Author contributions

ZZ: project host, data collection, and data analysis. YZ: data collection, paper writing, and data analysis. FF: data analysis and paper editing. AH and CR: data analysis and paper reviewing. All authors contributed to the article and approved the submitted version.

## Funding

This project was funded by the Hunan Provincial Social Science Foundation in China (Project Reference Number: XSP21YBC310).

## Conflict of interest

The authors declare that the research was conducted in the absence of any commercial or financial relationships that could be construed as a potential conflict of interest.

## Publisher’s note

All claims expressed in this article are solely those of the authors and do not necessarily represent those of their affiliated organizations, or those of the publisher, the editors and the reviewers. Any product that may be evaluated in this article, or claim that may be made by its manufacturer, is not guaranteed or endorsed by the publisher.
